# Identifying Alcohol Use Disorder With Resting State Functional Magnetic Resonance Imaging Data: A Comparison Among Machine Learning Classifiers

**DOI:** 10.3389/fpsyg.2022.867067

**Published:** 2022-06-10

**Authors:** Victor M. Vergara, Flor A. Espinoza, Vince D. Calhoun

**Affiliations:** Tri-Institutional Center for Translational Research in Neuroimaging and Data Science (TReNDS), Georgia State University, Georgia Institute of Technology, Emory University, Atlanta, GA, United States

**Keywords:** alcohol use disorder (AUD), functional network connectivity (FNC), fMRI, machine learning classifiers, resting state

## Abstract

Alcohol use disorder (AUD) is a burden to society creating social and health problems. Detection of AUD and its effects on the brain are difficult to assess. This problem is enhanced by the comorbid use of other substances such as nicotine that has been present in previous studies. Recent machine learning algorithms have raised the attention of researchers as a useful tool in studying and detecting AUD. This work uses AUD and controls samples free of any other substance use to assess the performance of a set of commonly used machine learning classifiers detecting AUD from resting state functional network connectivity (rsFNC) derived from independent component analysis. The cohort used included 51 alcohol dependent subjects and 51 control subjects. Despite alcohol, none of the 102 subjects reported use of nicotine, cannabis or any other dependence or habit formation substance. Classification features consisted of whole brain rsFNC estimates undergoing a feature selection process using a random forest approach. Features were then fed to 10 different machine learning classifiers to be evaluated based on their classification performance. A neural network classifier showed the highest performance with an area under the curve (AUC) of 0.79. Other good performers with similar AUC scores were logistic regression, nearest neighbor, and support vector machine classifiers. The worst results were obtained with Gaussian process and quadratic discriminant analysis. The feature selection outcome pointed to functional connections between visual, sensorimotor, executive control, reward, and salience networks as the most relevant for classification. We conclude that AUD can be identified using machine learning classifiers in the absence of nicotine comorbidity.

## Introduction

Alcohol use disorder (AUD) brings a series of social and health problems to individuals. Harmful alcohol consumption is one of the leading risk factors for population health in the world (World-Health-Organization, 2018). Alcohol consumption can create a series of health problems in important organs in the body such as the liver (Axley et al., 2019) and the brain (Rao and Topiwala, 2020). A series of neurocognitive problems are known to manifest in individuals with AUD (Caballeria et al., 2020) that might persist even after long periods of abstinence. These impairments are related to structural and functional effects of alcohol in the brain. Functional magnetic resonance imaging (fMRI) has recently proven a viable source of biomarkers for AUD (Zhu et al., 2018; Fede et al., 2019; Kamarajan et al., 2020) that deserves to be further explored and confirmed.

Problematic alcohol use is often assumed to be founded on the amount of alcohol consumption; however, statistical evidence shows this is not the case. Approximately 10% of the excessive drinkers is estimated to meet the criteria for AUD (Esser et al., 2014). Researchers thus look for a more comprehensive and reliable indicator of AUD. Screening tools for AUD are based on self-reported items for alcohol tolerance, withdrawal, impaired control, and unsuccessful attempts to cut down within the past month (American Psychiatric Association, 2013). Other instruments seem to agree with DSM-5. Large sensitivity and specificity for DSM-5 AUD with the Alcohol Use Disorders Identification Test (AUDIT) at the 8 or 9 AUDIT threshold (Hagman, 2016). Despite the existence of AUD tools that goes beyond the amount of alcohol consumed, there is still a need to test their validity with cautionary use recommendations advised for regular application (Baggio and Iglesias, 2020).

An important topic in AUD is the use of *in vivo* neuroimaging tools that can detect the existence of biomarkers in the brain of alcohol consumers (Fritz et al., 2019). Detriments to white and gray brain have been detected *via* computed tomography and structural magnetic resonance imaging. Biochemical changes in the brain related to alcohol have been quantified using magnetic resonance spectroscopy. Alcohol induced changes in neurotransmitters have been found using positron emission tomography. The quest for a more integral framework for AUD has recently highlighted the role of aberrant neurocircuitry related to alcohol consumption (Voon et al., 2020). A complete foundation to understand alcohol related neuropsychological impairments must consider the varied set of structural abnormalities and brain dysfunctions observed in the brain (Le Berre et al., 2019). Dysfunctional connectivity is commonly assessed from resting state (task free experiment) fMRI data where the status of connections among separate brain areas differs from those in a healthy brain. Aberrant neurocircuitry has been detected through fMRI in resting state experiments as a promising biomarker for relapse (Camchong et al., 2013). Evidence suggests that alcohol produces a larger number of dysfunctional connections compared to other commonly used substances including nicotine and cannabis (Vergara et al., 2018). This last piece of evidence underlines the viability of using functional connectivity as a source of information to detect AUD that can complement existing techniques relying on self-reported items.

The use of functional connectivity for assessment of AUD requires methods that can be applied to individual subjects. Machine learning classifiers (MLCs) using functional connectivity as discriminatory features have proved useful in several brain illnesses such as traumatic brain injury and schizophrenia (Yang et al., 2010; Vergara et al., 2017b). For the AUD perspective, MLCs can take an fMRI scan from the brain of a subject, designated as the testing sample, and estimate its AUD or non-AUD status within a certain accuracy and error estimates. The most common way this technology is configured consists on using a set of known AUD and non-AUD populations, known as the training sample, and estimate a series of parameters that MLCs use to classify any novel testing sample (Mak et al., 2019). One of the most explored MLC techniques in AUD is random forest (Tin Kam, 1998). Random forest decides based on the outcomes of several decision trees (a flow chart-like structure where each node represents a decision). Several studies have used random forest to evaluate the feasibility of functional connectivity in discriminating AUD from non-AUD subjects. In Kamarajan et al. (2020), a small set of 30 AUD and 30 non-AUD populations were used successfully with an accuracy of 76.67%. The most important features, reported in the Kamarajan study, were AUD hyperconnectivity at prefrontal cortex, parietal areas, anterior and posterior cingulate, and AUD hypoconnectivity in fronto-parietal and fronto-temporal regions. In Zhu et al. (2018), a larger set of 96 subjects was employed with a performance between 87.0 and 90.5%. Zhu and co-authors identified important features included the executive control network (ECN) encompassing areas of the anterior cingulate, frontal, and parietal lobes; and a reward network (RN) composed of subcortical areas including putamen, thalamus, nucleus accumbens and caudate. These outcomes provide strong evidence for the use of functional connectivity and MLCs as a complementary technique besides self-reported methods such as the DSM-5 and AUDIT questionnaires.

This study addresses two issues of current fMRI-AUD literature. The first issue to address is the existence of confounding nicotine use in the samples. Both Zhu et al. (2018) and Kamarajan et al. (2020) studies exhibit a significantly strong difference in nicotine use in the AUD sample compared to the non-AUD. Although alcohol seems to produce a larger number of connectivity abnormalities than nicotine (Vergara et al., 2017a,^2018^), it is not fully clear whether achieved classification performance was biased by nicotine or not. The second issue is the limitation of the MLC techniques considered. There are other MLCs available that could provide better performance in AUD classification than random forest but have not been included in the analysis. The experimental design of this work considers cohorts free of nicotine use and any other substance abuse. Also, age and sex have been matched between AUD and non-AUD populations. The main aim of this study is to provide evidence for MLC success in identifying AUD in an isolated alcohol use dimension. The results are important to either validate or disprove alcohol involvement on the performance of MLCs formerly reported in the existing literature. The second important aim pursue an exploratory evaluation of available MLCs providing a valuable comparative baseline when selecting specific classifiers for future AUD applications.

## Methods

### Subjects

Samples were drawn from a larger cohort scanned using the same fMRI protocol at The Mind Research Network in Albuquerque, New Mexico, United States. All participants provided informed consent according to the Declaration of Helsinki and the institutional guidelines at the University of New Mexico. Samples included 51 AUD subjects (34.6 ± 11.2 years old with 18 females) and 51 sex and age matched non-AUD samples. A demographics summary is available in Table 1. Subject exclusion criteria included nicotine use, brain injury, brain-related medical problems, and bipolar or psychotic disorders. A urinalysis test rejected the use of any drug including marijuana. AUD status was determined by applying the Alcohol Use Disorder Identification Test (AUDIT) (Saunders et al., 1993). AUD subjects were required to go through breathalyzer prior to participation and stop drinking at least 24 h before fMRI scanning. AUDIT scores (20.25 ± 7.9) ranged from 11 to 39. No AUDIT scores were available for the non-AUD group, but their non-AUD status was determined using the Structured Clinical Interview for DSM-IV-TR Axis I Disorders, Research Version, Patient Edition (SCID-I/P) (First et al., 2002). No DSM-IV data was available in the AUD group. Previous studies confirmed that DSM’s non-AUD corresponds with an AUDIT score less than nine (Hagman, 2016), settling the criterion for non-AUD in the current work. All control (non-AUD) subjects reported no use of nicotine or marijuana. Absence of nicotine addiction was confirmed using the Fagerström Questionnaire (Fagerström, 1978) where all samples scored zero.

**TABLE 1:** Demographics.

	AUD			Non-AUD		
		
	Males	Females	*t*-test males females	Males	Females	*t*-test males females
Number	33	18		33	18	
Age	35.5 (11.5)	33.1 (11.0)	*p* > 0.49	35.3 (11.8)	33.2 (11.0)	*p* > 0.51
AUDIT	20.0 (7.7)	20.7 (8.6)	*p* > 0.78	N/A	N/A	
FTND	0	0		0	0	

*Non-AUD status was determined using the DSM-IV. AUDIT is not available (N/A) for non-AUD.*

*FTND, Fagerström Test for Nicotine Dependence.*

### Functional Magnetic Resonance Imaging Data

Five minutes of eyes-open resting state data were collected on a 3T Siemens TIM Trio (Erlangen, Germany) scanner. Echo-planar EPI sequence images (TR = 2,000 ms, TE = 29 ms, flip angle = 75°) were acquired with an 8-channel head coil. Volumes consisted of 33 axial slices (64 × 64 matrix, 3.75 × 3.75 mm^2^, 3.5 mm thickness, 1 mm gap). Data were preprocessed using the statistical parametric mapping software (SPM)^1^ (Friston, 2003), including slice-timing correction, realignment, co-registration, spatial normalization, and transformation to the Montreal Neurological Institute (MNI) standard space. The DVARS method (Power et al., 2012) was used to find spike regressors where the root mean square (RMS) head movement exceeded 3 standard deviations. Time courses, with a size of 145-time steps, were orthogonalized with respect to (i) linear, quadratic, and cubic trends; (ii) the six realignment parameters, and (iii) realignment parameters derivatives. Realignment parameters were regressed out of the functional magnetic resonance imaging (fMRI) data and then smoothed using a FWHM Gaussian kernel of size 6 mm.

Infomax group independent component analysis (Calhoun and Adali, 2012), available through the Group ICA fMRI Toolbox (GIFT),^2^ was then applied to all 102 fMRI images. The GIFT toolbox implementation applied an ICASSO algorithm (Himberg et al., 2004) with 10 repetitions to verify the consistency of the group ICA result. Following previous fMRI classification studies (Vergara et al., 2017b), a set of 70 components was obtained from the group ICA analysis. Out of the 70 components, a total of 34 were selected as resting state networks (RSNs) based on frequency content and visual inspection. Supplementary Figure 1 shows the spatial content of included RSNs and Supplementary Table 1 contains the MNI coordinates.

Time courses of all RSNs were filtered using a fifth order Butterworth filter with band width of [0.01, 0.15] Hz. Resting state functional network connectivity (rsFNC) was estimated using the Fisher’s-Z transformed Pearson correlation coefficient. Each rsFNC was subject to an age and sex linear model and the model residual were used as classification features. A total of 561 (34*33/2) rsFNC features were available to feed into the machine learning classification workflow. For ease of analysis and illustration, selected RSNs were organized in nine domains (groups of RSNs) as reward network (RN; thalamus and putamen), auditory (auditory temporal areas), cerebellum, sensorimotor (supplementary motor area, pre- and post- central areas), visual (occipital, fusiform, cuneus, calcarine, and lingual giri), executive network (ECN; fronto-parietal RSNs), salience (insula), default mode network (DMN; ventromedial prefrontal, posterior and anterior cingulate), and language (several temporal areas).

### Comparing Machine Learning Methods

The set of considered rsFNC features were fed to a set of classification algorithms including Random Forest, Logistic Regression, Nearest Neighbors, Support Vector Machine (SVM) (Linear SVM and RBF SVM), Gaussian Process, Decision Tree, Neural Network (multilayer perceptron), AdaBoost, Naive Bayes, and quadratic discriminant analysis (QDA). These classification algorithms are available in python using the scikit library.^3^ A quick description of each of these algorithms is available in Supplementary Table 2. We selected this set of MLCs given their availability to the research community through the scikit library.

**FIGURE 1:**
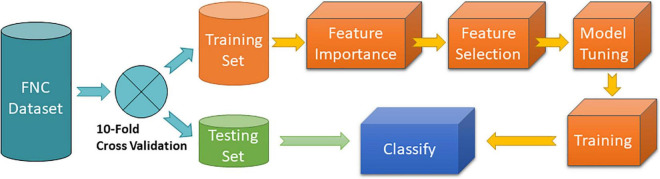
MLC 10-fold cross validation procedure. Feature importance was implemented using the random forest algorithm. Feature selection was programmed to include features with highest importance. Model tuning and training consisted of a grid search of different hyperparameters of the specific MLC algorithm tested.

In this work, we estimated MLC performance using a strict approach where testing samples do not participate on finding the optimal MLC model. The procedure is illustrated in Figure 1. A 10-fold cross validation strategy was used to partition the data into separate testing and training data sets. The training set was subject to several processes necessary to find the optimal MLC model including feature importance assessment, feature selection, model tuning and training. The trained MLC was then used to classify samples using the testing set. This procedure guarantees that no information of the test samples leaks into the MLC training.

Feature importance assessment was performed using random forest as this method has been selected in previous studies (Zhu et al., 2018; Kamarajan et al., 2020). Feature selection was achieved by selecting the top best features ranked by their importance value. A range of several numbers referred to as levels of feature selection were considered following a similar approach as that in Zhu et al. (2018). The first level used 10% of the most important features (56 in total). The second level included the 20% most important, then 30%, etc., until all 100% of features were included. A 10-fold cross validation loop was performed for each feature selection level and MLC hyperparameter tuning. Model tuning and training consisted of running the training samples for different hyperparameter settings of the MLC *via* a grid search procedure. The optimal model was selected using the maximum average area under the curve (AUC) metric, which measures the discriminatory ability of a binary classifier to differentiate between classes. Averages and standard deviations were calculated based on the 10 cross validated outcomes.

### Comparing Statistical Tests and Machine Learning Outcomes

**FIGURE 2:**
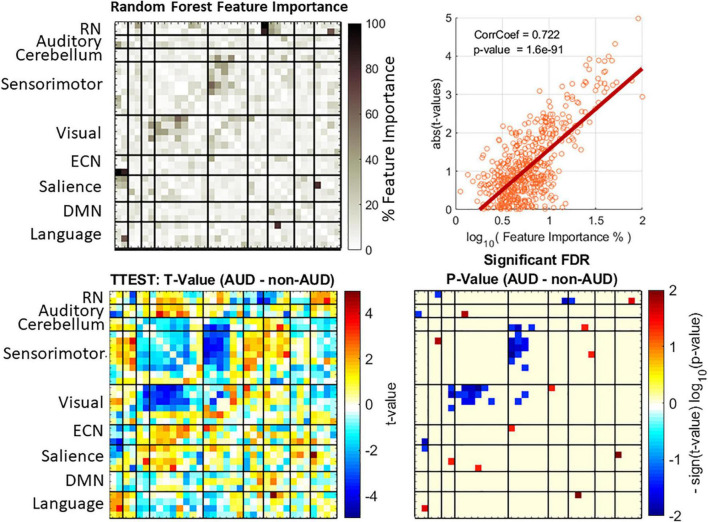
Feature importance and group difference assessments. Feature importance values were obtained from random forest (top left plot). Displayed feature importance % is the normalized average over the 10 iterations of the cross validation. Group differences were evaluated with t-statistics test (bottom left plot) and the *p*-values corrected using the false discovery rate (FDR) method. A strong correlation exists between the two approaches (top right plot).

Previous statistical analyses of AUD reported significant group differences in rsFNC that allowed making biological interpretation of the results (Vergara et al., 2017a). Although a motion related variance was addressed during data preprocessing, we tested group difference of a motion variance measure known as forward displacement (Power et al., 2012) using a two-sample *t*-test with a non-significant *p*-value of 0.28. To make consistent interpretations and comparisons with machine learning outcomes, we report significant group differences (AUD—non-AUD) in Figure 2. A total of 561 *t*-tests were performed (one for each rsFNC feature) and the statistical significance corrected using false discovery rate.

## Results

### Feature Importance and Functional Network Connectivity

All MLCs were subject to the same procedure in Figure 1 with 10 different feature importance assessments, one for each fold iteration. The mean feature importance was calculated as a representative of the whole cross validation. Normalized mean feature importance (divided over the maximum) is shown in Figure 2 displayed in a percent scale. Figure 2 also shows the correlation between group differences represented as the absolute value of the *t*-values and the logarithm of feature importance estimates. We used the absolute *t*-value because the *t*-value magnitude is a good indicator of classification relevance as it has been made evident by previous studies using *t*-value feature selection (Vergara et al., 2017b). In this work we preferred to use random forest for feature important assessment as a technique that requires fewer assumptions than the t-distribution statistics, however, the two methods might be correlated. Results in Figure 2 show that feature importance and *t*-values are correlated at 0.72 with a significant *p*-value of 1.6e-97. Important rsFNC features are concentrated in a cluster of visual-sensorimotor hypoconnectivity in AUD. The RN is also hypoconnected to some auditory, ECN and salience RSNs. Hyperconnectivity was found in RN-language, auditory-sensorimotor, cerebellum-salience, sensorimotor-salience, visual-ECN and salience-language. These results are similar to previous rsFNC results (Vergara et al., 2017a,^2018^).

### Classification Outcomes

**FIGURE 3:**
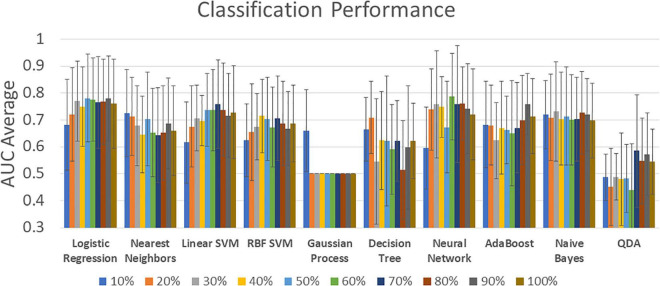
Classification performance for all classifiers and all feature selection levels. Percentage labels indicate the number of included rsFNC features. The complete set of classification results can be found in Supplementary Table 3.

Figure 3 shows a summary of the 10-fold cross validated AUC average classification performances. Other metrics such as Sensitivity (true positive rate), Specificity (true negative rate), and F1 are included in Supplementary Table 3. The model tuning step from Figure 1 was performed using a grid search and the considered hyperparameter can be found in Supplementary Table 4. The highest AUC obtained was 0.79 for the neural network classifier with 336 features (60%). Similar results were found in Logistic Regression with a max AUC of 0.78 for 50% features selected. The next best result was found in linear SVM with 0.76 at 70% features selection. However, the AUC standard deviation is large and there is no significant difference between most of the results. The exceptions are the two worst performers Gaussian Process and QDA classifiers exhibiting significantly lower AUC scores also not significantly different from chance. The Gaussian Process classifier has a visible lack of classification performance since most of the results stayed at 0.50 except for the lowest number of features 10% with an AUC of 0.66.

**FIGURE 4:**
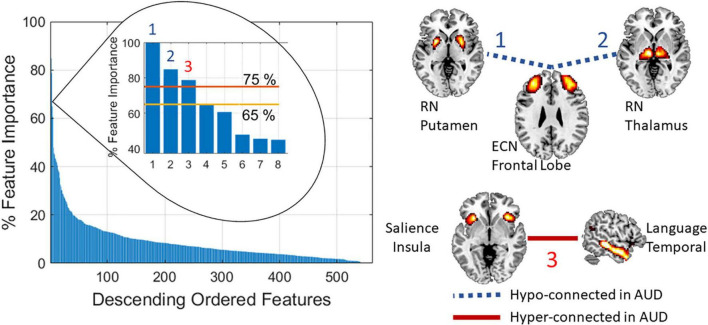
The three features with highest importance (>75%) are indicated by the numbers 1, 2, and 3. These features point to RN-ECN and Salience-Language connectivity.

The results in Figure 3 explored the effect of different number of features. The best results pointed to 336 features (60%), but analyzing all these features might result overwhelming. Figure 2 showed evidence on where to find the most important features among which a considerable cluster points to the connectivity between visual and sensorimotor domains. We made a further analysis to find the most important features and the results are illustrated in Figure 4. The three most important features indicated in Figure 4 were selected given their feature importance metric to be larger than 75% while all other features exhibit an importance below 65%. These three features relate to significant ECN-RN hypoconnectivity and salience-language hyperconnectivity in AUD and corresponds to the three darkest point (largest feature importance) in Figure 2.

## Discussion

Machine learning classification under a fully controlled alcohol-only experiment was the focus of this study making it the first report of its kind, as far as the authors are aware. A common co-morbid addiction in substance use disorders is nicotine, but non-smoking samples were carefully selected among AUD participants in addition to the exclusion of any other addictive substance. The sample used in this experiment is thus difficult to find but necessary for a valid evaluation of the utility of functional connectivity in diagnosing AUD. Experimental design reduced the possibility of using testing data information when assessing performance (avoiding data leakage) compared to previous implementations on alcohol data (Vergara et al., 2017b; Zhu et al., 2018; Kamarajan et al., 2020). We have also tested a variety of widely used classifiers comparing their classification performance in using functional connectivity from AUD subjects to provide important information for future experimental research and future applications.

The neural network classifier achieved the highest performance, as displayed in Figure 3. In general, most MLCs achieved similar performance. SVM results were proficient which is consistent with previous MLCs reports from different illnesses (Vergara et al., 2017b; Steardo et al., 2020; Rodriguez et al., 2021). Logistic regression has been used in AUD classification studies with high performance which is similar to the outcome observed in Figure 3 (Fede et al., 2019; Mu et al., 2020). Naïve Bayes and Nearest Neighbors have also been considered in functional connectivity and these MLCs were proficient in the current AUD study (Jahromy et al., 2019). Two MLC results stand out as non-advisable due to their low performance. Gaussian Process classifiers have been shown to work well on functional connectivity data (Challis et al., 2015), however, our observations show the predictions as indistinguishable from classification by chance (AUC mode of 0.500). QDA has been previously used in functional connectivity, but with low classification performance in concordance with our observations (McMenamin and Pessoa, 2015).

In fMRI studies, as well as in most classification frameworks, feature selection is used to control for the overfitting problem. When an algorithm is overfitting, the solution is based on features that might be irrelevant for general group separation, leading to underperforming problems handling new samples. Another problem of using suboptimal features is the observable detriment of classification performance (Du et al., 2018). Results illustrated in Figure 3 show the number of features affecting the maximum performance of proficient classifiers. A high AUC score can be interpreted as an optimal selection of relevant features. This result indicates a good strategy for an optimal feature selection. Of all considered classifiers in Figure 3, neural networks exhibited the most advantageous features.

Results pointed to important hypoconnectivity (negative *t*-values in Figure 2) in the visual and sensorimotor domains. This hypoconnectivity between visual and sensorimotor areas has been previously related to alcohol drinking with cuneus, postcentral, supplementary motor area, fusiform, and lingual gyri more frequently appearing in case of nicotine absence (Vergara et al., 2017a,^2018^). Among other observations, a previous seed-based study confirms the importance of the relationship between sensorimotor and visual networks in AUD (Muller-Oehring et al., 2015). Notice that features with largest importance for classification also point to significant rsFNC group differences. Figures 2, 4 illustrates this relationship indicating the role of the RN, ECN, language, and salience domains. In agreement with Zhu’s report (Zhu et al., 2018), our results indicate the large relevance of connectivity between subcortical areas of the RN (basal ganglia and thalamus) and the ECN in classifying AUD. Reduced connectivity in ECN, basal ganglia, and visual areas was also previously reported with significant associations to years of drinking and severity of alcohol problems (Weiland et al., 2014). The next most important feature was the connectivity between the salience network represented by the anterior insula and the language network located in the temporal lobe. These connections are the most important ones as indicated in Figure 4.

Known resting state dysfunctions in AUD related to the temporal gyrus are scarcely observed in the literature. However, insula and temporal gyri suffer from gray matter reduction, among many other structures, linked to AUD (Yang et al., 2016; Rolland et al., 2020). Continuing with fMRI data, task-based studies found important effects of AUD in the insula cortex and the temporal gyrus including hyperconnectivity as a reaction to alcohol stimuli (Strosche et al., 2021), abnormal neural activity (Karch et al., 2015), abnormal brain function in verbal working memory (Park et al., 2011), and indications of neurobiological correlates to cue-reactivity (Zeng et al., 2021). Comparative observations from task-based studies mentioned in this paragraph, previous AUD classification reports (Zhu et al., 2018; Fede et al., 2019; Kamarajan et al., 2020), and those in the current work suggests that insula and temporal gyrus fMRI abnormalities linked to AUD are more prominent during exteroceptive brain function of task execution. However, our study was not designed to deal with this hypothesis that will have to be tested by research work in the future. Following one of the key premises of our study, it is possible that enforcing absence of nicotine use in the current sample was the condition allowing for alcohol related rsFNC contrast to emerge as an important feature.

One noticeable item in the current results is the little contribution of DMN connectivity for the AUD classification. However, the DMN is an important area affected in AUD (Muller-Oehring et al., 2015). Another study also pointed to DMN as an important contributor for AUD classification, but the reason might be the use of DMN seeds to perform the classification experiment (Kamarajan et al., 2020). There are two experimental conditions that could have led to this network’s classification contribution difference. First, the absence of nicotine in the sample. A multi-substance use report showed that visual-sensorimotor hypoconnectivity is a characteristic of alcohol users, but DMN areas were more affected by nicotine use (Vergara et al., 2017a). The same study found ECN areas such as the inferior parietal lobule and salience areas such as the insula as affected, but only in subjects that consume both nicotine and alcohol. The second experimental condition to consider is the withdrawal time. In this work, subjects were scanned within 24 h of sobriety, in the (Kamarajan et al., 2020) study within 5 days, and the (Zhu et al., 2018) study with at least a week of abstinence averaging 26.9 days. Abstinence time could be a factor that changes the specific brain networks involved in classification. Visual-sensorimotor networks might be strongly affected shortly after alcohol consumption overshadowing effects in other networks. In addition to contributions from salience, basal ganglia, DMN, and ECN, a study using machine learning to predict AUDIT found that features from visual, sensorimotor, auditory, and language networks are very important (Fede et al., 2019). A study by Camchong et al. delineated the importance of visual networks in early abstinence as a feature for predicting recovery outcomes (Camchong et al., 2013). Our results consider absence of nicotine influence and a very short abstinence period.

The procedure used in this work was stricter in dealing with data leakage problems when compared to other publications. While this is an advantage of the current analysis, it does make it difficult to compare with previously published results. For comparison, we repeated the analysis using a procedure similar to that one used in Zhu et al. (2018). The outcomes are described with detail in the Supplementary Analysis document accompanying this manuscript. In summary, relaxing leakage concerns leads to AUC values as high as 0.89 for the neural network classifier which is the same best classifier for the stricter procedure. A noticeable difference is that other classifiers achieved similar performance to neural networks including Logistic Regression, Nearest Neighbors, Naïve Bayes and both SVM kernels. Another detail is that AUC tends to decrease as the number of features selected increases. These observations replicate those similarly reported in Zhu et al. (2018), where including the 10% best features achieved an accuracy of 0.87, but this performance decreased to 0.72 when all features were included. Another similar study achieved 0.76 accuracy after including fMRI features restricted to the DMN, plus neuropsychological and impulsiveness scores (Kamarajan et al., 2020). The procedure was based on the random forest method and did not implement cross validation relying solely on the classifier outcome. Our and Zhu’s results agrees that AUD identification features should not be restricted to the DMN. The observation in the current work suggests that DMN connectivity is of lower importance and better classification is achieved by including other areas in the brain.

The current results explored alcohol use as the only dimension. Limiting our experimental design to alcohol validated AUD detection *via* MLC providing evidence that alcohol, and not comorbid substance use, induces rsFNC changes that can be used as classification features. However, this work did not address comorbidity, it rather focused on its absence. Full MLC performance under comorbidity will be topic of future research with different sample cohort and multi-label classification techniques that can differentiate more than two groups. Another limitation in this study is the different methods to determine AUD status. This work used different criterion to determine alcohol use based on evidence from comparative studies showing similarities in AUDIT and DSM outcomes (Dawson et al., 2012; Foxcroft et al., 2015). There is also a likely alcohol dependence in the AUD group given its mean AUDIT of 20 (Saunders et al., 1993) and a likely absence of dependence in the non-AUD samples. Since no extra stratification of AUD has been considered, there is no reason to doubt samples identification. However, the existence of false positives and negatives are a problem in any test and the results of this work are limited to these alcohol use instruments. Along with mentioned statistical problems is the problem that MLC usually requires a large number of training samples which is limited. Although our experiment only included 102 samples the outcomes are important as an alcohol only study. Future studies in comorbidity should use larger sample sets to better characterize comorbidity and stratification of use seeking behaviors.

In sum, this work has strongly selected samples to focus on AUD—non-AUD differentiation with matched demographics and absence of nicotine comorbidity. Results indicate that connectivity between salience and reward (basal ganglia and thalamus) networks is one of the most important features for detecting AUD. Second in importance is the connectivity between visual and sensorimotor areas. Despite the careful variable control, the limitation of our results strives in the general need for a larger number of samples to train machine learning algorithms. Finding larger sets of training samples with a good strategy for controlling unrelated variables is a challenge that will have to be addressed in future studies.

## Data Availability Statement

The original contributions presented in this study are included in the article/Supplementary Material, further inquiries can be directed to the corresponding author.

## Ethics Statement

The studies involving human participants were reviewed and approved by the Institutional Review Board of the University of New Mexico. The patients/participants provided their written informed consent to participate in this study.

## Author Contributions

VV and VC were responsible for the data collection and project planning. VV oversaw the project execution including algorithm design and implementation. FE performed the machine learning analysis and created the final report. All authors contributed to the article and approved the submitted version.

## Conflict of Interest

The authors declare that the research was conducted in the absence of any commercial or financial relationships that could be construed as a potential conflict of interest.

## Publisher’s Note

All claims expressed in this article are solely those of the authors and do not necessarily represent those of their affiliated organizations, or those of the publisher, the editors and the reviewers. Any product that may be evaluated in this article, or claim that may be made by its manufacturer, is not guaranteed or endorsed by the publisher.

## Footnotes

1
http://www.fil.ion.ucl.ac.uk/spm

2
http://trendscenter.org/software/gift/

3
https://scikit-learn.org/stable/
