# Supplementary material for: Identifying Alcohol Use Disorder With Resting State Functional Magnetic Resonance Imaging Data: A Comparison Among Machine Learning Classifiers
Source: Front Psychol. 2022 Jun 10;13:867067. doi: 10.3389/fpsyg.2022.867067 (PMC9226579; doi:10.3389/fpsyg.2022.867067)
Supplement: Supplementary file 2 [file Data_Sheet_2.PDF]

## Supplementary Table II

Summary description of each machine learning classifier employed in this study.

| **Classifier Name** | **Python Scikit Classifier Description (**Ref: https://scikit-learn.org/stable/supervised_learning.html#supervised-learning) |
|---|---|
| Random Forest (RF) | In random forests (see **RandomForestClassifier** and **RandomForestRegressor** classes), each tree in the ensemble is built from a sample drawn with replacement (i.e., a bootstrap sample) from the training set. Furthermore, when splitting each node during the construction of a tree, the best split is found either from all input features or a random subset of size max features. (See the parameter tuning guidelines for more details).<br><br>The purpose of these two sources of randomness is to decrease the variance of the forest estimator. Indeed, individual decision trees typically exhibit high variance and tend to overfit. The injected randomness in forests yield decision trees with somewhat decoupled prediction errors. By taking an average of those predictions, some errors can cancel out. Random forests achieve a reduced variance by combining diverse trees, sometimes at the cost of a slight increase in bias. In practice the variance reduction is often significant, hence yielding an overall better model. |
| Logistic Regression (LR) | Logistic regression, despite its name, is a linear model for classification rather than regression. Logistic regression is also known in the literature as logit regression, maximum-entropy classification (MaxEnt) or the log-linear classifier. In this model, the probabilities describing the possible outcomes of a single trial are modeled using a logistic function.<br><br>Logistic regression is implemented in **LogisticRegression**. This implementation can fit binary, One-vs-Rest, or multinomial logistic regression with optional ℓ1, ℓ2 or Elastic-Net regularization. |
| K-Nearest Neighbors (KNN) | Neighbors-based classification is a type of *instance-based learning* or *non-generalizing learning*: it does not attempt to construct a general internal model, but simply stores instances of the training data. Classification is computed from a simple majority vote of the nearest neighbors of each point: a query point is assigned the data class which has the most representatives within the nearest neighbors of the point.<br><br>scikit-learn implements two different nearest neighbors classifiers: **KNeighborsClassifier** implements learning based on the k nearest neighbors of each query point, where k is an integer value specified by the |

| | |
|---|---|
| | user. **RadiusNeighborsClassifier** implements learning based on the number of neighbors within a fixed radius r of each training point, where r is a floating-point value specified by the user.<br><br>The k-neighbors classification in **KNeighborsClassifier** is the most commonly used technique. The optimal choice of the value k is highly data-dependent: in general, a larger k suppresses the effects of noise, but makes the classification boundaries less distinct. |
| Support Vector Machine [linear kernel (linearSVM), rbf kernel (rbfSVM)] | **Support vector machines (SVMs)** are a set of supervised learning methods used for classification, regression and outliers detection. SVMs decision function (detailed in the Mathematical formulation) depends on some subset of the training data, called the support vectors. Some properties of these support vectors can be found in attributes support_vectors_, support_ and n_support_.<br><br>For classification problems, the **SVC** class is capable of performing binary and multi-class classification on a dataset. As other classifiers, **SVC**, take as input two arrays: an array X of shape (n_samples, n_features) holding the training samples, and an array y of class labels (strings or integers), of shape (n_samples). |
| Gaussian Process (GP) | The **GaussianProcessClassifier** implements Gaussian processes (GP) for classification purposes, more specifically for probabilistic classification, where test predictions take the form of class probabilities.<br><br>GaussianProcessClassifier places a GP prior on a latent function f, which is then squashed through a link function to obtain the probabilistic classification. The latent function f is a so-called nuisance function, whose values are not observed and are not relevant by themselves. Its purpose is to allow a convenient formulation of the model, and f is removed (integrated out) during prediction. GaussianProcessClassifier implements the logistic link function, for which the integral cannot be computed analytically but is easily approximated in the binary case. |
| Decision Tree (DT) | **Decision Trees (DTs)** are a non-parametric supervised learning method used for classification and regression. The goal is to create a model that predicts the value of a target variable by learning simple decision rules inferred from the data features. A tree can be seen as a piecewise constant approximation.<br><br>In classification, **DecisionTreeClassifier** is a class capable of performing binary and multi-class classification on a dataset. As with other classifiers, **DecisionTreeClassifier** takes as input two arrays: an array X, sparse or dense, of shape (n_samples, n_features) holding the training samples, and an array Y of integer values, shape (n_samples,), holding the class labels for the training samples. After being fitted, the model can then be used to predict the class of samples. |

| | |
|---|---|
| Neural Network (NN) | **Multi-layer Perceptron (MLP)** is a supervised learning algorithm that learns a function f(·): Rm→Ro by training on a dataset, where m is the number of dimensions for input and o is the number of dimensions for output. Given a set of features X=x1,x2,...,xm and a target y, it can learn a non-linear function approximator for either classification or regression. It is different from logistic regression, in that between the input and the output layer, there can be one or more non-linear layers, called hidden layers.<br><br>The class **MLPClassifier** implements a multi-layer perceptron (MLP) algorithm that trains using Backpropagation. MLP trains on two arrays: array X of size (n_samples, n_features), which holds the training samples represented as floating point feature vectors; and array y of size (n_samples,), which holds the target values (class labels) for the training samples. |
| AdaBoost (AB) | AdaBoost can be used both for classification and regression problems. The core principle of AdaBoost is to fit a sequence of weak learners (i.e., models that are only slightly better than random guessing, such as small decision trees) on repeatedly modified versions of the data. The predictions from all of them are then combined through a weighted majority vote (or sum) to produce the final prediction. |
| Naive Bayes (NB) | Naive Bayes methods are a set of supervised learning algorithms based on applying Bayes' theorem with the "naive" assumption of conditional independence between every pair of features given the value of the class variable.<br><br>In spite of their apparently over-simplified assumptions, naive Bayes classifiers have worked quite well in many real-world situations, famously document classification and spam filtering. They require a small amount of training data to estimate the necessary parameters.<br><br>Naive Bayes learners and classifiers can be extremely fast compared to more sophisticated methods. The decoupling of the class conditional feature distributions means that each distribution can be independently estimated as a one-dimensional distribution. This in turn helps to alleviate problems stemming from the curse of dimensionality.<br><br>On the flip side, although naive Bayes is known as a decent classifier, it is known to be a bad estimator, so the probability outputs from predict_proba are not to be taken too seriously. |
| Quadratic Discriminant Analysis (QDA) | Quadratic Discriminant Analysis (**QuadraticDiscriminantAnalysis**) is a classic classifier with a quadratic decision surface. This classifier is attractive because it has closed-form solutions that can be easily computed, are inherently multiclass, have proven to work well in practice, and have no hyperparameters to tune. |
