# Supplementary material for: Identifying Alcohol Use Disorder With Resting State Functional Magnetic Resonance Imaging Data: A Comparison Among Machine Learning Classifiers
Source: Front Psychol. 2022 Jun 10;13:867067. doi: 10.3389/fpsyg.2022.867067 (PMC9226579; doi:10.3389/fpsyg.2022.867067)
Supplement: Supplementary file 4 [file Data_Sheet_4.PDF]

Supplementary Table IV

Hyperparameters used in the model tuning step of Figure 1.

All pipelines of the grid search used the function StandardScaler() to standardize the data.

Classifier	Python Scikit Classifier Instantiation	Grid Search
Random Forest (RF)	RandomForestClassifier (random_state=42)	n_estimators = [5, 10, 15, 50, 100, 200, 500], max_depth = [2, 4, 8, 16, 32, None]
Logistic Regression (LR)	LogisticRegression(max_iter=10000, tol=1e-08, random_state=1)	C = [0.01, 0.05, 0.1, 0.5, 1.0, 5.0, 10.0], solver = ['lbfgs', 'liblinear']
K-Nearest Neighbors (KNN)	KNeighborsClassifier()	n_neighbors = [3, 5, 7, 9, 11], weights = ['uniform', 'distance'], p = [1, 2]
Support Vector Machine (linear kernel, linearSVM)	SVC(kernel="linear", max_iter=10000, tol=1e-08, random_state=1)	C = [0.01, 0.05, 0.1, 0.5, 1.0, 5.0, 10.0], Gamma = ['scale', 'auto']
Support Vector Machine (rbf kernel, rbfSVM)	SVC(kernel='rbf', max_iter=10000, tol=1e-08, random_state=1))	C = [0.01, 0.05, 0.1, 0.5, 1.0, 5.0, 10.0], gamma = ['scale', 'auto']
Gaussian Process (GP) Kernel = [1.0 * RBF(1.0)]	GaussianProcessClassifier(random_state=1)	No parameters to search.
Decision Tree (DT)	DecisionTreeClassifier(random_state=1))	criterion = ['gini', 'entropy'], max_depth = [2, 3, 4, 5, 6, 7, 8], max_features = [2, 3, 4, 5, 6, 8, 10, 20, 30, 100]
Neural Network (NN)	MLPClassifier(random_state=1, max_iter=500)	hidden_layer_sizes = [1, 5, 10, 15, 20]
AdaBoost (AB)	AdaBoostClassifier(random_state=1)	n_estimators = [10, 20, 30, 40, 50]
Naive Bayes (NB)	GaussianNB()	var_smoothing': [1e+0, 1e-01, 1e-02, 1e-03, 1e-04, 1e-05, 1e-06, 1e-07, 1e-08, 1e-09]
Quadratic Discriminant Analysis (QDA)	QuadraticDiscriminantAnalysis()	No parameters to search.
