# Supplementary material for: Identifying Alcohol Use Disorder With Resting State Functional Magnetic Resonance Imaging Data: A Comparison Among Machine Learning Classifiers
Source: Front Psychol. 2022 Jun 10;13:867067. doi: 10.3389/fpsyg.2022.867067 (PMC9226579; doi:10.3389/fpsyg.2022.867067)
Supplement: Supplementary file 5 [file Data_Sheet_5.docx]

Supplementary Analysis

Identifying Alcohol Use Disorder with Resting State fMRI Data: A Comparison Among Machine Learning Classifiers

Victor M. Vergara PhD, Flor A. Espinoza PhD, and Vince D. Calhoun PhD

Tri-Institutional Center for Translational Research in Neuroimaging and Data Science (TReNDS)

Georgia State University, Georgia Institute of Technology, and Emory University,

Atlanta, GA, USA

Corresponding Author:

Victor M. Vergara

Tri-institutional center for Translational Research in Neuroimaging and Data Science (TReNDS)

55 Park Place, Atlanta GA 30303

Telephone: 404-413-5488 Fax: 404-413-5124

Email: [vvergarascience@gmail.com](mailto:vvergarascience@gmail.com)

# Introduction

Previous studies using machine learning classifiers (MLCs) have reported high classification accuracy using a random forest classifier. We considered accuracies from two very similar studies including Kamarajan’s study achieving 76% (Kamarajan et al., 2020) and Zhu’s study ranging from 72% with no feature elimination up to 87% with 90% of features eliminated. Samples from these two studies exhibited comorbid smoking leading to one important premise in this work that nicotine might have affected the AUD classification outcomes. The current set of 102 samples are nicotine free determined using the Fagerström score (Fagerström, 1978). Our first attempt was to use a similar procedure to that in Kamarajan’s and Zhu’s studies where each classification step was performed in sequence. The sequence we used is similar to that found in Zhu’s Figure 3 (Zhu, Du, Kerich, Lohoff, & Momenan, 2018). This initial procedure followed the next illustration.


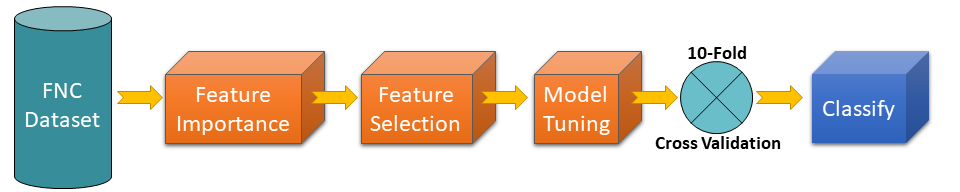


The sequential steps procedure has a high probability of exhibiting data leakage. This is information regarding testing samples leaking and inflating the classification. This problem can be argued true since feature importance, selection and tuning used all samples and did not separate training and testing sets, a procedure implied in the cross validation. The procedure was slightly changed as shown in Figure 1 in the main manuscript to avoid data leakage. However, we provide results from the sequential steps to include a fair comparison with results from previous publications.

# Methods

The methods described in the main manuscript are almost all valid in the current supplement, except for the sequential classifier evaluation.

# Results

Averages and variances were based on the 10 iterations of the applied cross validation. The highest area under the curve (AUC) obtained was 0.89 for the neural network classifier with 56 features (10 %). In general, the more features included the higher is the detriment to AUC assessments. This tendency has been previously observed in machine learning classification of AUD (Zhu et al., 2018). Similar results were found in Logistic Regression, Nearest Neighbors, Naïve Bayes and both SVM kernels. Another trend is the decrease of AUC variance as the number of features also decreases. This is further evidence that removed features contribute to nuisance variability in the classification models. The worst performers were the Gaussian Process and QDA classifiers. QDA showed a different trend related to the number of features. The Gaussian Process classifier has a visible lack of classification performance since most of the results stayed at 0.50 except for the lowest number of features at 0.73. QDA improved the performance as the number of features increased, but it never performs better than the other algorithms.


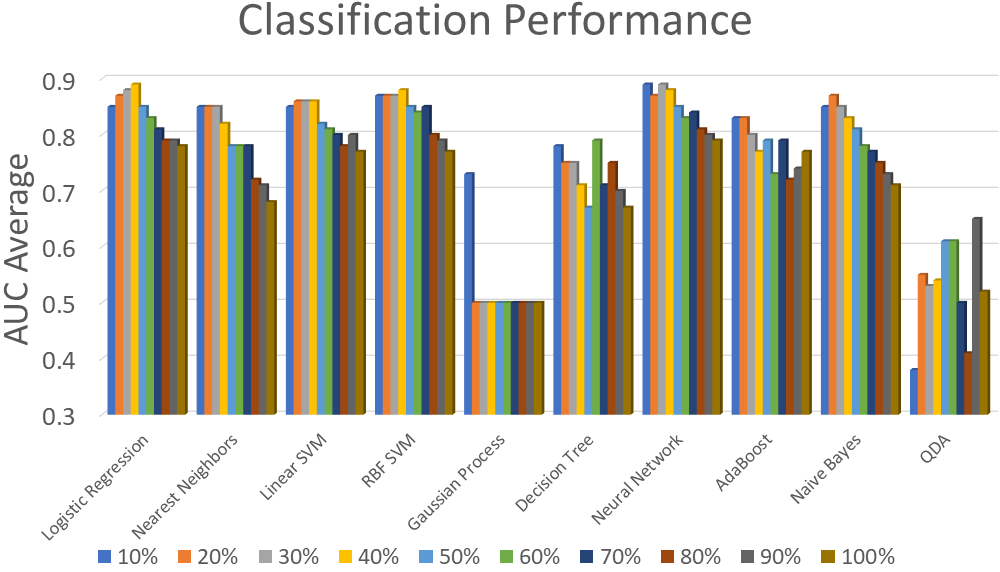


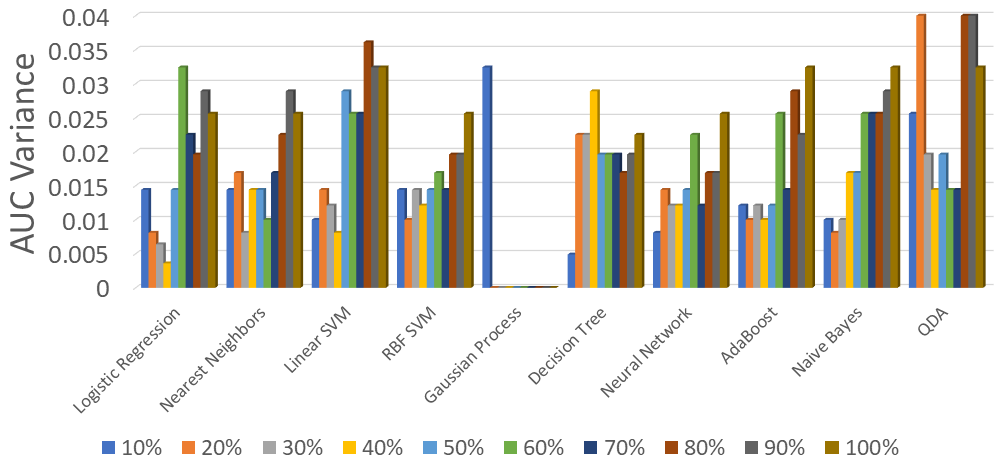


# Discussion

The neural network classifier achieved the highest performance for a low number of features (10% and 30% of the total) but was similar to other classifiers for the optimal number of features. In general, SVM methods achieved one of the highest performances which is in line with previous machine learning classification reports from different illnesses (Rodriguez et al., 2021; Steardo et al., 2020; Vergara, Mayer, Damaraju, Kiehl, & Calhoun, 2017). Logistic regression has been used in classification studies with high performance according to the performance observed in Figure 2 (Fede, Grodin, Dean, Diazgranados, & Momenan, 2019; Mu et al., 2020). Naïve Bayes and Nearest Neighbors have also been considered in functional connectivity (Jahromy, Bajoulvand, & Daliri, 2019). Gaussian Process classifiers have been shown to work well on functional connectivity data (Challis et al., 2015). This result is in line with a high sensitivity to noisy inputs in the Gaussian Process classifier (Villacampa-Calvo, Zaldivar, Garrido-Merchán, & Hernández-Lobato, 2020). QDA has been previously used in functional connectivity, but with low classification performance in concordance with our observations (McMenamin & Pessoa, 2015).

Compared to the two previous studies (Kamarajan et al., 2020) and (Zhu et al., 2018), the current results show that controlling for nicotine variance and selecting an appropriate MLC can deliver similar or better classification performance. The best AUC in our comparative analysis was slightly better 89% than the 87% accuracy obtained in (Zhu et al., 2018) for a similar feature reduction. The results presented in this supplement do not make a strict control for data leakage, but the numerical assessments can be better compared to the two mentioned studies that might suffer from similar issues. The analysis in the main manuscript used a stricter leakage control procedure and the numerical performance are prone to be lower, but with more realistic translational assessment.

# References

Challis, E., Hurley, P., Serra, L., Bozzali, M., Oliver, S., & Cercignani, M. (2015). Gaussian process classification of Alzheimer's disease and mild cognitive impairment from resting-state fMRI. *Neuroimage, 112*, 232-243. doi:10.1016/j.neuroimage.2015.02.037

Fagerström, K.-O. (1978). Measuring degree of physical dependence to tobacco smoking with reference to individualization of treatment. *Addictive Behaviors, 3*(3-4), 235-241. doi:10.1016/0306-4603(78)90024-2

Fede, S. J., Grodin, E. N., Dean, S. F., Diazgranados, N., & Momenan, R. (2019). Resting state connectivity best predicts alcohol use severity in moderate to heavy alcohol users. *Neuroimage Clin, 22*, 101782. doi:10.1016/j.nicl.2019.101782

Jahromy, F. Z., Bajoulvand, A., & Daliri, M. R. (2019). Statistical algorithms for emotion classification via functional connectivity. *J Integr Neurosci, 18*(3), 293-297. doi:10.31083/j.jin.2019.03.601

Kamarajan, C., Ardekani, B. A., Pandey, A. K., Kinreich, S., Pandey, G., Chorlian, D. B., . . . Porjesz, B. (2020). Random Forest Classification of Alcohol Use Disorder Using fMRI Functional Connectivity, Neuropsychological Functioning, and Impulsivity Measures. *Brain Sci, 10*(2). doi:10.3390/brainsci10020115

McMenamin, B. W., & Pessoa, L. (2015). Discovering networks altered by potential threat ("anxiety") using quadratic discriminant analysis. *Neuroimage, 116*, 1-9. doi:10.1016/j.neuroimage.2015.05.002

Mu, J., Chen, T., Quan, S., Wang, C., Zhao, L., & Liu, J. (2020). Neuroimaging features of whole-brain functional connectivity predict attack frequency of migraine. *Hum Brain Mapp, 41*(4), 984-993. doi:10.1002/hbm.24854

Rodriguez, C. I., Vergara, V. M., Davies, S., Calhoun, V. D., Savage, D. D., & Hamilton, D. A. (2021). Detection of prenatal alcohol exposure using machine learning classification of resting-state functional network connectivity data. *Alcohol, 93*, 25-34. doi:10.1016/j.alcohol.2021.03.001

Steardo, L., Jr., Carbone, E. A., de Filippis, R., Pisanu, C., Segura-Garcia, C., Squassina, A., . . . Steardo, L. (2020). Application of Support Vector Machine on fMRI Data as Biomarkers in Schizophrenia Diagnosis: A Systematic Review. *Front Psychiatry, 11*, 588. doi:10.3389/fpsyt.2020.00588

Vergara, V. M., Mayer, A. R., Damaraju, E., Kiehl, K. A., & Calhoun, V. (2017). Detection of Mild Traumatic Brain Injury by Machine Learning Classification Using Resting State Functional Network Connectivity and Fractional Anisotropy. *J Neurotrauma, 34*(5), 1045-1053. doi:10.1089/neu.2016.4526

Villacampa-Calvo, C., Zaldivar, B., Garrido-Merchán, E. C., & Hernández-Lobato, D. (2020). Multi-class Gaussian Process Classification with Noisy Inputs. *arXiv preprint arXiv:2001.10523*.

Zhu, X., Du, X., Kerich, M., Lohoff, F. W., & Momenan, R. (2018). Random forest based classification of alcohol dependence patients and healthy controls using resting state MRI. *Neurosci Lett, 676*, 27-33. doi:10.1016/j.neulet.2018.04.007
