# Supplementary figures and images for: Identifying Alcohol Use Disorder With Resting State Functional Magnetic Resonance Imaging Data: A Comparison Among Machine Learning Classifiers
Source: Front Psychol. 2022 Jun 10;13:867067. doi: 10.3389/fpsyg.2022.867067 (PMC9226579; doi:10.3389/fpsyg.2022.867067)

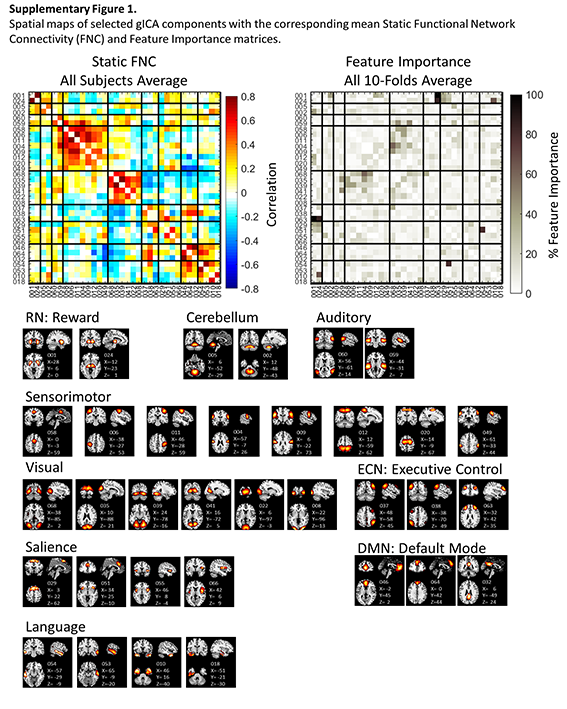

Supplement: Supplementary file 6 [file Image_1.TIF]
